# Validity of Self-perceived and Clinically Diagnosed Gingival Status among 12–15-year-old Children in Indonesia

**DOI:** 10.31372/20190402.1033

**Published:** 2019

**Authors:** Robbykha Rosalien, Frida Avianing Isnanda Saragih, Ary Agustanti, Febriana Setiawati, Diah Ayu Maharani

**Affiliations:** a Oral Epidemiology and Clinical Studies in Dentistry Cluster, Faculty of Dentistry, Universitas Indonesia, Indonesia; b Department of Preventive and Public Health Dentistry, Faculty of Dentistry, Universitas Indonesia, Indonesia

**Keywords:** dental plaque index, gingival index, sensitivity and specificity, self-perception

## Abstract

A few studies have revealed the self-perceived gingival status using questionnaires among children. Perceived health is a crucial factor that has an impact on quality of life. The objective of the study was to assess self-perceived and clinically diagnosed gingival status among children in Indonesia. This was a cross-sectional study of 494 schoolchildren (aged 12–15 years). Periodontal status was recorded using the gingival index (GI) and plaque index (PI) based on the World Health Organization standards. Data were collected through a brief visual, non-invasive clinical oral examination and a self-administered questionnaire. The sensitivity and specificity of self-perceived assessment were calculated using normative assessment as the gold standard. This study showed that self-perceived need for dental treatment showed the highest sensitivity (86% using PI and 85% using GI) and self-perceived swollen gums showed the highest specificity (89% using PI and 88% using GI) for clinically diagnosed plaque (PI cut-off value: 0.74) and gingival problems (GI cut-off value: 0.51). In conclusion, both self-perceived variables showed significant discordance between their respective sensitivity and specificity. Self-perceived information is at a higher-level unawareness that does not reflect the current gingival status. Thus, public health strategies are needed to improve the awareness of better oral health among children by promoting, empowering, and advocating.

## Introduction

Periodontal diseases and dental caries are highly prevalent chronic conditions across the world (Jin et al., 2016). Oral diseases and conditions can affect individuals across the life course, and have a negative impact on quality of life (Thomson & Broder, 2018). Periodontal disease is one of the most prevalent diseases in Indonesia, especially among children. In a study conducted in Jakarta, 68% schoolchildren aged 12 years were found to have gingivitis (Adiatman et al., 2016). Gingivitis is a periodontal condition that is a local response developed from dental plaque, whose clinical signs include gingival bleeding. Poor oral hygiene and behavior will impact on gingival status (Rosalien et al., 2018). Therefore, improvement in oral health conditions is a key imperative which also requires valid measurement to diagnose and identify the diseases.

The comparison between clinical (normative) and self-perceived assessment are important to evaluate the efficacy diagnostic data of oral health status (Maharani et al., 2019). Traditionally, the clinical or normative assessment has been predominantly used in dentistry to measure oral health status and treatment needs (Alves, de Andrade, & Vettore, 2015). However, for a large country such as Indonesia, it is difficult to undertake oral health survey annually (Maharani, 2009a). In a time of limited resources, as is currently the case in Indonesia, the use of subjective indicators as screening instruments provide a rapid and inexpensive way to annually evaluate oral health in Indonesia (Maharani, 2009a).

It has been suggested that self-perceived oral health and subjective perceptions regarding oral health status play a key role on whether people will seek oral health care and improve the awareness for better oral health. On the other hand, lack of perceptions regarding oral health need constitutes an important barrier to the awareness and utilization of oral health care services (Maharani, 2009b). Self-perceived assessment has been found to be a useful measurement to assess an individual’s oral health status (Ueno, Zaitsu, Ohara, Wright, & Kawaguchi, 2015). Although studies about self-perception exist, only few studies have evaluated the validity of self-perceived compared to clinically diagnosed gingival status among children in Indonesia. Hence, this study aimed to analyze the sensitivity and specificity of the self-perceived oral hygiene status and the gingival status clinically diagnosed among children 12–15 years of age.

## Methods

### Study Design

A cross-sectional study was carried out among junior high school students in Jakarta. Six schools were randomly selected out of the 287 officially listed schools. The schools were located across Central Jakarta, East Jakarta, South Jakarta, and North Jakarta. Subsequently, all children in the target age group of 12–15 years old and within the selected schools were invited to take part in this research.

### Sample Size Calculation

Sample size was calculated considering 80% statistical power and *α* level of 0.05. Considering an effect of 1.2 owing to cluster sampling and a further 15% to account for nonresponse, 597 students were invited to participate in the study. However, 71 participants did not participate in both the clinical examination as well as the questionnaire, while 32 schoolchildren either did not attend clinical examination or did not complete the questionnaire.

### Ethical Approval

Ethical approval was obtained from the Research Ethics Committee of Faculty Dentistry, Universitas Indonesia (No. 17/Ethical Approval/FKGUI/IV/2017).

### Data Collection

Data collection was conducted during April–July 2017. Information sheets and consent forms were distributed and obtained from parents and children to obtain consent and assent. The process was facilitated by the teachers. Data were collected through a brief visual non-invasive clinical oral examination and administration of a questionnaire. This study involved two dentists and two interviewers who were adequately trained to maintain standardization in all study procedures. Kappa scores for gingival index (GI) and plaque index (PI) were 0.70 and 0.71, respectively. The oral examination focused on measurement of GI and PI. Both indices were determined using dental probe and dental mirrors (Petersen, Baez, & World Health Organization, 2013). PI and GI were used to assess oral hygiene and gingival health of the children, with scores as follows: PI = ‘0’ no plaque, ‘1’ plaque visible on probing only, ‘2’ visible plaque; GI = ‘0’ no bleeding, ‘1’ minimal to moderate bleeding, ‘2’ widespread or spontaneous bleeding. Both in PI and GI recording, if the measurement was unable to be taken on the tooth, such as missing tooth, the measurement was not recorded (Adiatman et al., 2016). Perceived oral health was defined by the individual’s self-assessed oral health status. Each participant was required to answer eight questions. The questions pertained to the individual self-perceived oral health condition, their satisfaction related to their teeth, their self-perceived treatment needs, crooked teeth condition, bleeding gums, swollen gums, dental plaque and/or tartar, and bad breath. The sensitivity (the proportion of people with disease who have a positive result) and specificity (the proportion of people without the disease who have a negative result) of various questions in the self-perceived assessment questionnaire were calculated using clinical examination as the gold standard (Habib, Alalyani, Hussain, & Almutheibi, 2015).

### Data Analysis

Data were checked, entered, and cleaned in SPSS 20 (SPSS, Inc., Chicago, IL, USA), which was further used for statistical analysis. Statistically significant levels were chosen at *p* < 0.05.

## Results

Overall, 494 students (82.7% response rate) completed the consent and assent form, underwent clinical examination, and completed the questionnaire. The students consisted of 59% girls and 41% boys. Almost all students have plaque and gingivitis, with mean PI and GI less than 1, which corresponds to relatively good oral hygiene and to mild gingivitis category (Table 1).

**Table 1:** The Prevalence of PI > 0 and GI > 0, and Mean Values of PI and GI (n = 494)

	*n* (%)	Mean (SD)
Plaque index	492 (99.6)	0.74 (0.52)
Gingival index	473 (95.7)	0.51 (0.55)

Further, the cut-off points for each index were based on the mean value. PI scores from 0 to 0.74 was considered good oral hygiene status, and scores above 0.74 were categorized as lower oral hygiene status. Whereas GI scores between 0 and 0.51, and more than 0.51 were categorized as good and poor gingival health status, respectively. The sensitivity and specificity of different self-perceived variables in the questionnaire were calculated using the cut-off values for PI and GI (Table 2). Self-perceived need for oral treatment showed the highest sensitivity, while its specificity was low. On the other hand, swollen gums showed the highest specificity, while its sensitivity was low. Similar results were obtained for both oral treatment needs and swollen gums when assessed against the gingival index.

**Table 2:** Sensitivity and Specificity of Self-perceived Gingival Health Using 0.74 as the Cut-off Plaque Index Score and Using 0.51 as the Cut-off Gingival Index Score

	GI = 0.51				PI = 0.74		
Variable	SS (%)		SP (%)		SS (%)		SP (%)
Opinion regarding dental health condition	67		53		67		54
Satisfaction with dental health condition	64		43		66		45
Oral treatment needs	85		18		86		18
Crooked teeth	55		47		59		49
Bleeding gums	45		73		42		72
Swollen gums	23		88		23		89
Plaque and/or tartar	63		54		61		54
Bad breath	61		47		61		48

SS: sensitivity; SP: specificity; GI: gingival index; PI: plaque index.

## Discussion

This study found that self-perception of oral hygiene and gingival health did not show good agreement with clinical assessed plaque and gingival index. The self-perceived need for oral health treatment showed good sensitivity for both indices; however, the specificity value for both indices were low. Opinion regarding dental health condition showed moderate sensitivity and specificity. Perception of oral health could differ by lifestyles, health behaviors, diet, and socioeconomic status (Ueno et al., 2015). Although clinical data might be the preferred measure for surveillance of oral health assessment, the cost and resources for acquiring self-report measures may be more attainable. Thus, surveillance of oral health based on self-perceived measures can be used in the interim to broaden surveillance where resources for clinically based surveillance are scarce (Eke et al., 2013).

Bleeding gums that are easily noticed by children showed higher sensitivity and specificity for relatively lower oral hygiene and gingival status. These findings are consistent with those reported by Baser, Germen, Erdem, Issever, and Yalcin (2014). Children’s perception of swollen gums, which is a clinical sign of gingivitis, was captured by the question: The question, “In the past three months, have you ever had swollen gums?” showed a lower sensitivity as compared to its specificity. These finding give a valuable insight for public health services that children do not feel their oral health compromised until they are affected by certain clinical signs or certain symptoms. Similar results were found in Japanese study, where the participants may not have sought dental treatment until the symptoms occurred (Ueno et al., 2015). The method of diagnosis for oral hygiene and gingival health, based on a full-mouth examination, may be impractical for use in population-based studies for reasons of time and cost. Although self-reported oral health have demonstrated potential bias in estimating plaque and gingival index, full-mouth examination might still be impractical for large studies. Therefore, screening for oral health disease using self-reported oral health information might still be useful in large epidemiologic surveys (Litaker, 2014).

The findings suggest that children seem unable to detect properly that whether they are affected by oral hygiene problem; they tend to overestimate their oral health condition. Furthermore, the level of oral health perception and perceived need have been found to influence oral health seeking behavior and is related to the utilization of dental services for early detection and prevention of oral health diseases (Maharani, 2009b; Maharani & Rahardjo, 2012). The limitation of this study is in the generalizability. This study sample is representative of the population in Jakarta, the capital city of Indonesia. Further study need to be in a large sampling that represents Indonesia’s adolescents overall. The present study highlights several important issues. It has been noted that schoolchildren have less ability to recognize oral hygiene and gingival health problems and that they might be having low level of awareness of the need for dental treatment as well. However, financial constraints, culture, and poor accessibility to health services might also be responsible for the high prevalence of oral health diseases.

## Conclusion

None of the sensitivity and specificity of self-perceived questionnaire using plaque and gingival indices showed high for both variables. Oral health promotion strategies must be developed to improve the awareness and better oral health.

## Declaration of Conflicting Interests

The authors declared no potential conflicts of interest with respect to the research, authorship, and/or publication of this article.

## Funding

This study was supported by Universitas Indonesia.

## References

[R1] Adiatman M., Yuvana A. L., Nasia A. A., Rahardjo A., Maharani D. A., & Zhang S. (2016). Dental and periodontal status of 5 and 12 year old children in Jakarta and it’s satellite cities. *Journal of Dentistry Indonesia*, 23, 5–9. 10.14693/jdi.v23i1.982

[R2] Alves F. N. M., de Andrade C. L. T., & Vettore M. V. (2015). Planning oral health care using the sociodental approach and the index of family living conditions: A cross-sectional study in Brazilian adolescents. *BMC Research Notes*, 8, 588 10.1186/s13104-015-1564-3 26486730 PMC4618128

[R3] Baser U., Germen M., Erdem Y., Issever H., & Yalcin F. (2014). Evaluation of gingival bleeding awareness by comparison of self-reports and clinical measurements of freshman dental students. *European Journal of Dentistry*, 8, 360–365. 10.4103/1305-7456.137649 25202217 PMC4144135

[R4] Eke P. I., Dye B. A., Wei L., Slade G. D., Thornton-Evans G. O., Beck J. D., ... Genco R. J. (2013). Self-reported measures for surveillance of periodontitis. *Journal of Dental Research*, 92, 1041–1047. 10.1177/0022034513505621 24065636

[R5] Habib A., Alalyani M., Hussain I., & Almutheibi M. S. (2015). Brief review on sensitivity, specificity and predictivities. *IOSR Journal of Dental and Medical Sciences*, 14, 66–68. 10.9790/0853-14456468

[R6] Jin L. J., Lamster I. B., Greenspan J. S., Pitts N. B., Scully C., & Warnakulasuriya S. (2016). Global burden of oral diseases: Emerging concepts, management and interplay with systemic health. *Oral Diseases*, 22, 609–619. 10.1111/odi.12428 26704694

[R7] Litaker M. S. (2014). Self-reported measures may be useful in surveillance for periodontitis. *Journal of Evidence-Based Dentistry Practice*, 14, 67–69. 10.1016/j.jebdp.2014.04.020 24913530

[R8] Maharani D. A. (2009). Inequity in dental care utilization in the Indonesian population with a self-assessed need for dental treatment. *Tohoku Journal of Experimental Medicine*, 218, 229–239. 10.1620/tjem.218.229 19561394

[R9] Maharani D. A. (2009). Perceived need for and utilization of dental care in Indonesia in 2006 and 2007: A secondary analysis. *Journal of Oral Science*, 51, 545–550. 10.2334/josnusd.51.545 20032606

[R10] Maharani D. A., Kurniawan J., Agustanti A., Rosalien R., Rahardjo A., & Cavalcanti A. L. (2019). Diagnostic validity of self-perceived dental caries in Indonesian young adolescents aged 12–15 years. *Pesquisa Brasileira em Odontopediatria e Clinica Integrada*, 19, e4543 10.4034/PBOCI.2019.191.04

[R11] Maharani D. A., & Rahardjo A. (2012). Is the utilisation of dental care based on need or socioeconomic status? A study of dental care in Indonesia from 1999 to 2009. *International Dental Journal*, 62, 90–94. 10.1111/j.1875-595X.2011.00095.x 22420478 PMC9374974

[R12] Petersen P. E., Baez R. J., & World Health Organization. (2013). *Oral health surveys: Basic methods* (5th ed.) World Health Organization.

[R13] Rosalien R., Hutami D. F., Agustanti A., Septalita A., Adiatman M., & Maharani D. A. (2018). Gingival health status of 12-year-old school children in Jakarta: A cross-sectional study. *Makara Journal of Health Research*, 22, 95–98. 10.7454/msk.v22i2.9736

[R14] Thomson W. M., & Broder H. L. (2018). Oral-health–related quality of life in children and adolescents. *Pediatric Clinics of North America*, 65, 1073–1084. 10.1016/j.pcl.2018.05.015 30213350

[R15] Ueno M., Zaitsu T., Ohara S., Wright C., & Kawaguchi Y. (2015). Factors influencing perceived oral health of Japanese middle-aged adults. *Asia–Pacific Journal of Public Health*, 27, NP2296-304 10.1177/1010539511428352 22186388

